# Atrial flutter with aberrant conduction in a patient taking amphetamine salts and caffeine

**DOI:** 10.3402/jchimp.v1i4.10663

**Published:** 2012-01-26

**Authors:** Marc Mugmon

**Affiliations:** Department of Medicine, Union Memorial Hospital, Baltimore, MD, USA

## Abstract

A case is presented of a man with a life-threatening tachyarrhythmia precipitated by ingested substances.

A 26-year-old Caucasian man with a history of attention-deficit hyperactivity disorder (ADHD) treated with mixed amphetamine salts developed severe lightheadedness and palpitations while running on a treadmill. He had taken his usual dose that day, as well as a 5-hour energy drink (containing 138 mg of caffeine), which he usually did once daily.

His past history was unremarkable. There was no prior cardiac history and no history of drug or alcohol abuse.

The paramedic crew discovered a wide complex tachycardia at a rate of 300 beats per minute (Fig. 1) and he underwent cardioversion at the scene because of hemodynamic instability. Sinus rhythm was immediately restored, his blood pressure was normalized, and his symptoms were resolved. Comprehensive metabolic profile, including electrolyte and thyroid panel, was unremarkable. Toxicology screen was negative. A urine screen for mixed amphetamine salts was not obtained. Peak troponin was 2.14, leading to coronary angiography, that revealed normal coronary arteries. Left ventriculography revealed normal systolic function and no apical ballooning was noted.

**Fig. 1:**
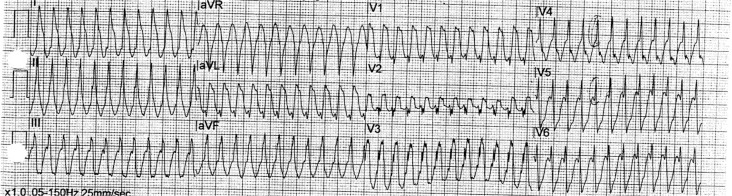
Electrocardiogram demonstrating atrial flutter with 1:1 conduction.

Electrophysiologic testing was attempted but was aborted due to multiple recurrent episodes of atrial flutter, atrial fibrillation, and other atrial arrhythmias. He required cardioversion during the EP study because of atrial flutter. A brief attempt at ablation was made at that time but was abandoned. He was placed on sotalol.

The next day, he underwent a second EP study, and atrial flutter with 1:1 conduction was demonstrated. Successful ablation of the cavatricuspid isthmus was performed and atrial flutter could not be induced.

Monitoring for several more days for amphetamine withdrawal symptoms revealed no further arrhythmias.

This patient had atrial flutter with 1:1 conduction and aberrant conduction, and this was confirmed during electrophysiologic testing. Initially, the treating personnel thought the rhythm to be ventricular tachycardia.

This patient's arrhythmias were presumed to have been due to a combination of caffeine use and amphetamine salts, in a setting of underlying enhanced atrioventricular (AV) conduction. Atrial arrhythmias, especially atrial fibrillation, have been associated with highly caffeinated beverage consumption in healthy adolescents (1). However, in a Danish registry of almost 48,000 participants, the risk of atrial fibrillation or flutter was not increased with caffeine consumption (2).

Amphetamines and other stimulant drugs used to treat ADHD are related to sympathomimetic amines that can have significant neurologic and cardiovascular stimulant effects. The Food and Drug Administration added a black box warning pointing out the possibility of ‘serious cardiovascular adverse events and sudden cardiac death reported with misuse.’

This patient did not appear to be misusing his ADHD medication, but it was suspected that the addition of caffeine in conjunction with enhanced AV conduction helped to precipitate a dangerous tachyarrhythmia.

Differentiating ventricular tachycardia from supraventricular tachycardia with aberrantly conducted beats can be difficult. Hemodynamic stability or instability is not a reliable differentiating characteristic because ventricular tachycardia may cause minimal symptoms in some patients, and atrial flutter with a rapid rate may lead to serious circulatory compromise in others. However, in case of doubt, any wide complex tachycardia should be assumed to be ventricular, and amiodarone should be administered or cardioversion should be performed. Electrophysiologic testing should be deferred until the patient is hemodynamically stable enough to undergo the procedure.

Conduction delay or block in the His-Purkinje system during antegrade conduction of impulses over the AV conduction system results in a wide, abnormal QRS. Features suggesting aberrant conduction are the presence of an initially narrow QRS portion and a right bundle branch block pattern. Features suggesting ventricular tachycardia include (1) the absence of an RS complex in the precordial leads and (2) AV dissociation. Other features of ventricular tachycardia outlined in a 1991 article by Brugada and colleagues are known as the Brugada Criteria (3).

In summary, this case demonstrates that therapy with stimulants for the treatment of ADHD, in combination with other cardioactive substances, can precipitate serious cardiac rhythm abnormalities in patients who may have underlying conduction disturbances. It also demonstrates that atrial flutter must be considered in a tachyarrhythmia when the rate is 300.
